# Association between physician adoption of a new oral anti-diabetic medication and Medicare and Medicaid drug spending

**DOI:** 10.1186/s12913-019-4520-4

**Published:** 2019-10-16

**Authors:** Ilinca D. Metes, Lingshu Xue, Chung-Chou H. Chang, Haiden A. Huskamp, Walid F. Gellad, Wei-Hsuan Lo-Ciganic, Niteesh K. Choudhry, Seth Richards-Shubik, Hasan Guclu, Julie M. Donohue

**Affiliations:** 1 Department of Health Policy and Management, Graduate School of Public Health, University of Pittsburgh, 130 DeSoto Street, Crabtree Hall A651, Pittsburgh, PA 15261 USA; 2 Department of Epidemiology, Graduate School of Public Health, University of Pittsburgh, 130 DeSoto Street, Crabtree Hall A651, Pittsburgh, PA 15261 USA; 3 Department of Medicine, School of Medicine, University of Pittsburgh, 200 Meyran Avenue, Suite 200, Pittsburgh, PA 15213 USA; 4 Department of Biostatistics, Graduate School of Public Health, University of Pittsburgh, Pittsburgh, PA 15261 USA; 5 Department of Health Care Policy, Harvard Medical School, 180 Longwood Avenue, Boston, MA 02115 USA; 6 Center for Pharmaceutical, Policy and Prescribing, Health Policy Institute, University of Pittsburgh, Pittsburgh, PA 15261 USA; 7 Center for Health Equity Research and Promotion, Veterans Affairs Pittsburgh Healthcare System, University Drive (151C), Pittsburgh, PA 15215 USA; 8 Department of Pharmaceutical Outcomes and Policy, College of Pharmacy, University of Florida, 1225 Center Drive, Gainesville, FL 32610 USA; 9 Division of Pharmacoepidemiology and Pharmacoeconomics, Department of Medicine, Brigham and Women’s Hospital and Harvard Medical School, 1620 Tremont Street, Suite 3030, Boston, MA 02120 USA; 10 College of Business and Economics, Lehigh University, Rausch Business Center, Room 465, 621 Taylor St, Bethlehem, PA 18015 USA; 11 Department of Statistics, School of Science, Istanbul Medeniyet University, Uskudar, 34700 Istanbul, Turkey; 12 Department of Health Policy and Management, Graduate School of Public Health, University of Pittsburgh, 130 DeSoto Street, Crabtree Hall A635, Pittsburgh, PA 15261 USA

**Keywords:** Prescription drugs, Physician behavior, Technology adoption, Medicare, Medicaid, Diabetes

## Abstract

**Background:**

In the United States, there is well-documented regional variation in prescription drug spending. However, the specific role of physician adoption of brand name drugs on the variation in patient-level prescription drug spending is still being investigated across a multitude of drug classes. Our study aims to add to the literature by determining the association between physician adoption of a first-in-class anti-diabetic (AD) drug, sitagliptin, and AD drug spending in the Medicare and Medicaid populations in Pennsylvania.

**Methods:**

We obtained physician-level data from QuintilesIMS Xponent™ database for Pennsylvania and constructed county-level measures of time to adoption and share of physicians adopting sitagliptin in its first year post-introduction. We additionally measured total AD drug spending for all Medicare fee-for-service and Part D enrollees (*N* = 125,264) and all Medicaid (*N* = 50,836) enrollees with type II diabetes in Pennsylvania for 2011. Finite mixture model regression, adjusting for patient socio-demographic/clinical characteristics, was used to examine the association between physician adoption of sitagliptin and AD drug spending.

**Results:**

Physician adoption of sitagliptin varied from 44 to 99% across the state’s 67 counties. Average per capita AD spending was $1340 (SD $1764) in Medicare and $1291 (SD $1881) in Medicaid. A 10% increase in the share of physicians adopting sitagliptin in a county was associated with a 3.5% (95% CI: 2.0–4.9) and 5.3% (95% CI: 0.3–10.3) increase in drug spending for the Medicare and Medicaid populations, respectively.

**Conclusions:**

In a medication market with many choices, county-level adoption of sitagliptin was positively associated with AD spending in Medicare and Medicaid, two programs with different approaches to formulary management.

## Background

There is substantial regional variation in prescription drug spending in the United States [1, 2], a finding that is consistent across different classes of drugs, patient populations, and health care payers (e.g. Medicare, VA) [1, 3–5]. Much of this variation is attributed to differences in the extent to which physicians prescribe brand name medications as opposed to generic medications, and not to differences in the volume of prescriptions filled, or to patient characteristics [1]. Regional differences in brand name drug prescribing are likely tied to regional differences in the speed with which physicians adopt new drugs. Studies have evidenced tremendous *physician-level* variation in adoption speed in several drug categories [6–10]; however, the association between *region-level* differences of physician adoption of newly introduced brand name drugs and prescription drug spending is still poorly understood.

Improving our understanding of how new drug adoption drives prescription drug spending is paramount for U.S. policy makers in the face of ever rising health care expenditures and an aging population that will likely increase demand for chronic disease medications. We examine the association between physician adoption and drug spending for diabetes for three reasons. First, diabetes is a progressive chronic disease that is increasing in prevalence and accounts for a large share of prescription drug and medical spending [11–13]. Second, there are multiple FDA approved anti-diabetic drugs available, with varying mechanisms of action, effectiveness, and prices. However, there is little evidence-based guidance for physicians on which medications to prescribe when augmenting therapy [14]. Third, the continual introduction of new brand name anti-diabetic drugs complicates physician decision-making and increases the potential for variation in new drug adoption.

Our study aimed to examine local variation in physician adoption of sitagliptin, a first-in-class oral glycemic lowering agent introduced in October 2006, and to investigate the association between physician adoption of sitagliptin and overall anti-diabetic drug spending in two large, and distinct, payer settings (Medicare and Medicaid). Sitagliptin was the first dipeptidyl peptidase-4 (DPP-4) inhibitor introduced to the market, but was not considered as a first-line treatment option. Therefore, sitagliptin represents the introduction of an expensive brand name drug, considered a moderately novel diabetes treatment, into a market that contained a large number of both generic and brand name treatment options, plus multiple, highly expensive, insulin alternatives [14]. Thus, investigating the role of physician adoption of sitagliptin can highlight how the entry of even one brand name drug in the midst of complex treatment options can influence physician decision making, high variability in new drug adoption, and overall drug spending.

## Methods

### Data sources

We conducted a cross-sectional analysis using data from three sources. First, we obtained Medicare claims and enrollment data from the Centers for Medicare & Medicaid Services (CMS) for all fee-for-service Medicare enrollees who were residents of Pennsylvania (PA) and also enrolled in a Part D plan for 2011 (*N* = 855,361). We obtained all medical claims (MEDPAR, outpatient, carrier, home health, hospice, DME) as well as the Part D Event (PDE) file, which contains prescription details such as drug name, fill date, National Drug Code (NDC), and the total amount paid to the pharmacy from all sources (plan and beneficiary). We obtained beneficiary enrollment dates, demographic information, and ZIP code of residence from the Medicare Beneficiary Summary Files.

Second, we obtained claims, encounter, and enrollment data on all fee-for-service and managed care PA Medicaid enrollees for 2011 (*N* = 1,127,123) from the Pennsylvania Department of Human Services (PADHS) through an intergovernmental agreement. Demographic information and eligibility status were obtained from the Medicaid enrollment file. Prescription drug claims contain information on the drug name, fill date, NDC, and the amount paid to the pharmacy. As we obtained Medicaid data directly from PADHS and not from CMS, we capture drug utilization and medical claims among Medicaid managed care enrollees who make up a majority (~ 75%) of enrollees in the state. PADHS requires comprehensive reporting of encounter data from the managed care plans with which it contracts so the data provide a reliable and valid measure of utilization among managed care enrollees [15].

Third, we obtained physician-level prescribing data from QuintilesIMS Xponent™ which directly captures > 70% of all US prescriptions filled in retail pharmacies, including all payers (Medicare, Medicaid fee-for-service, commercial insurance, cash, and uninsured). Xponent™ utilizes a patented proprietary projection method to represent 100% of prescriptions filled in these outlets and has been widely used by researchers to examine medication use patterns [9, 16–20]. Our Xponent™ data includes all physician prescribers practicing in PA during January 2007–December 2011.

### Physician study sample

We excluded those physicians who did not prescribe at least one anti-diabetic drug each quarter in 2007 (the first full year following sitagliptin’s introduction in October 2006) so that our physician study sample would include only physicians who were regularly seeing diabetes patients, and were thus eligible to adopt sitagliptin (See Additional file 1: Appendix A for list of anti-diabetic study drugs). To ensure that these physicians were then also continuously seeing patients post-sitagliptin’s introduction, without also conditioning specifically on sitagliptin prescribing, we further included only those physicians who prescribed > 1 drug each year (2008–2011) from the following widely used medication classes: anti-coagulants, anti-hypertensives, or statins. Physicians were assigned to one of PA’s 67 counties using the zip code of their primary practice location. Three small counties (Cameron, Forest, and Sullivan) had ≤2 providers prescribing anti-diabetic drugs in 2007 and were excluded from the analysis. The final study sample included 7614 physicians (See Additional file 1: Appendix B).

### Measures of physician adoption

Our key independent variables were first measured at the physician-level and then aggregated to the county-level. For each physician in our sample, we measured the first month sitagliptin was dispensed to one of their patients, consistent with previous studies measuring physician adoption of new drugs [21–23]. In order to capture both speed and extent of physician adoption of sitagliptin we then constructed two measures: 1) mean time (in months) to first sitagliptin prescription across all physicians in a county using 2007–2011 data, and 2) percent of physicians within a county prescribing sitagliptin at least once in 2007. For the first measure, we chose to allow a five year period for the study physicians to adopt sitagliptin, this is based on prior literature, which has found that the rate of physicians adopting a newly introduced drug plateaus between three and five years post-market introduction [9, 24]. Additionally, the latter measure was weighted by each physician’s total anti-diabetic prescription volume to give higher weight to physicians with high patient volumes:

$$ \frac{\sum \left(\frac{AD\  prescribing\ volum{e}_{physician}}{AD\  prescribing\ volum{e}_{county}}\ast \#\mathrm{physicians}\ \mathrm{prescribed}\ \mathrm{sitagliptin}\ \mathrm{in}\ 12\ \mathrm{months}\right)}{\# of\ physicians\ in\ county}. $$

We conducted a sensitivity analysis including a measure of the percent of physicians in each county adopting sitagliptin not weighted by prescribing volume, and found the results were qualitatively similar.

### Medicare and Medicaid study samples

We constructed separate study samples and conducted all analyses separately for Medicare and Medicaid (See Additional file 1: Appendix C1 and C2 for study sample construction). Since Medicare is the primary payer for beneficiaries who have dual eligibility in both Medicare and Medicaid, dual eligible beneficiaries were included in the Medicare study sample and excluded from the Medicaid sample. For both study samples, we included patients if they: had a continuous 12 months of enrollment in 2011, were ≥ 18 years old on January 1, 2011, were PA residents, filled ≥1 prescription for an anti-diabetic medication in 2011, and met the Chronic Condition Data Warehouse (CCW) Algorithm for diabetes [24, 25]. Additionally, because our study drug, sitagliptin, is not indicated for type I diabetes, we limited both study samples to those with type II diabetes. Individuals who met the CCW algorithm were identified as having type II diabetes if they filled at least one oral anti-diabetic medication in 2011, or if they filled only insulin during 2011 but had ≥50% of all inpatient and outpatient diabetes related claims coded with type II specific ICD-9 codes (250.× 0 or 250.× 2).

### Dependent variables: anti-diabetic drug spending

The dependent variables for our analyses were patient-level Medicare and Medicaid anti-diabetic prescription drug spending in 2011. For Medicare, total annual drug spending included both plan payment and beneficiary out-of-pocket spending. For Medicaid, total drug spending included the total plan payment amount, in the case of managed care enrollees, or state payment amount for fee-for-service enrollees. PA Medicaid does require small copayments of its members for some prescription drugs; however, diabetes medications are excluded [26, 27].

### Covariates

We included several patient-level variables known to be associated with anti-diabetic drug spending including demographic characteristics, eligibility category and/or type of enrollment status, and clinical factors [28]. Demographic factors include age, sex, and racial or ethnic group (white, black, Hispanic, or other race/ethnicity). For Medicare, enrollment status included indicators for dual eligibility with Medicaid, Part D low-income subsidy (LIS) status, and disability vs. age as reason for eligibility. For Medicaid eligibility, we included categorical variables indicating Temporary Assistance to Needy Families enrollment (TANF), General Assistance enrollment, or Supplemental Security Income enrollment. In Medicaid, we also controlled for whether an enrollee was in fee-for-service or Medicaid managed care. We constructed the Elixhauser co-morbidity index using medical claims as a proxy for overall health status [29]. Finally, we included an indicator of the type of anti-diabetic drug(s) used: oral agents only, injectable agents only (which included all insulins plus exenatide and liraglutide), or a combination of oral and injectable anti-diabetic drugs. As this is a claims based study, no clinical indicators of diabetes disease severity (e.g. hbA1C) were readily available, thus, this measure was included as a potential proxy of diabetes severity, as patients having more intensified treatment with injectable agents or a combination of oral and injectable agents, are likely to have longer disease duration or worse severity, and are more likely to have tried multiple different treatment options than patients on oral agents alone [14].

### Statistical analysis

**Fig. 1:**
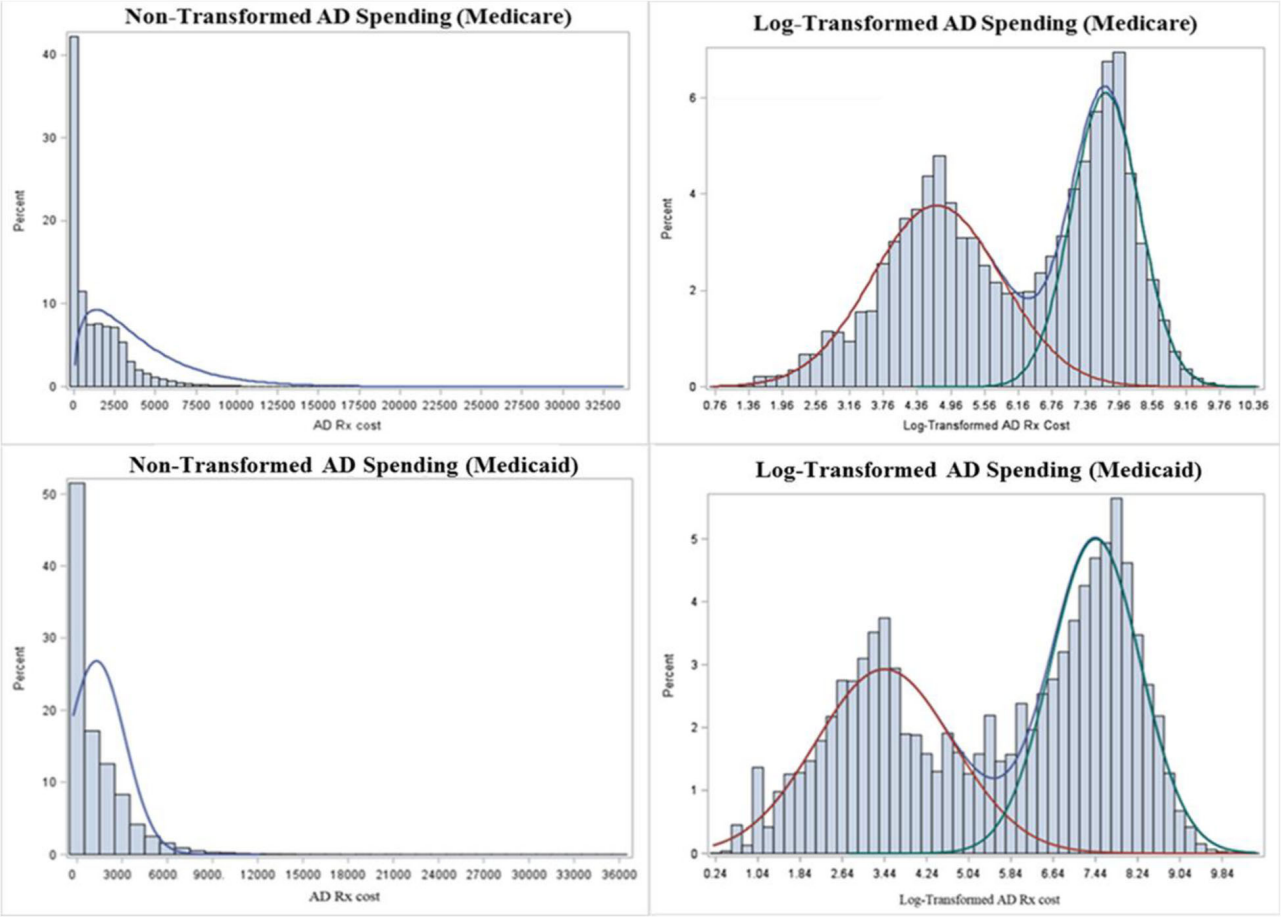
Raw and Log-Transformed Anti-Diabetic Drug Spending Distributions for the Medicare and Medicaid Study Samples (2011)

We first examined descriptive statistics for all study variables in both the Medicare and Medicaid samples. Means (SD) were used to describe all continuous variables and frequencies (percentage) were used to describe all categorical variables. After calculating the two adoption measures, we examined any county-level trends and patterns of these two measures. Second, we examined the distribution of the outcome anti-diabetic drug spending and found it to be highly skewed. After log transforming anti-diabetic drug spending, we found multiple modals of the transformed variable in both the Medicare and Medicaid study populations (Fig. 1). Therefore, we used finite mixture models to empirically identify the patient subgroups in both the Medicare and Medicaid study samples. Third, we fit the appropriate finite mixture model including the key explanatory adoption variables to investigate the association of regional physician adoption of sitagliptin with anti-diabetic drug spending. All covariates of interest were also included in the final models for adjustment.

### Latent subgroup identification

We used finite mixture models to empirically identify patient subgroups based on annual anti-diabetic drug spending. Finite mixture models use model-based posterior probabilities to assign individual observations to different subgroups (e.g. an observation will be assigned to the subgroup with the highest posterior subgroup membership probability) and can analytically capture unobservable heterogeneity in the different underlying subgroups. The true number of subgroups in a data set is unknown, and no gold standard exists in determining the “optimal” number of subgroups. The preferred method of model selection is through an iterative estimation where multiple models with different assumed numbers of subgroups, and no covariates, are fit. We fit models composed of one, two, or three subgroups and examined normal distributions, gamma distributions, or a combination of both normal and gamma distributions. The final number of subgroups, and selection of distributions, were selected based on the Bayesian information criterion (BIC) and the mean posterior probability values [30]. Applying these criteria, the two-component model consisting of two normal distributions had the best model fit for both Medicare and Medicaid (i.e., the lowest BIC and good classification according to high mean posterior probabilities) (Additional file 1: Appendix D1 and D2). For each individual subgroup, descriptive characteristics were inspected to examine potential patient features associated with group membership (Additional file 1: Appendix E).

### Association of Sitagliptin Adoption with anti-diabetic drug spending

The two-component finite mixture model, with county-level clustering, was then used to estimate the effect of county-level physician adoption of sitagliptin on patient drug spending in both Medicare and Medicaid. We hypothesized adoption speed (time to first prescription) to be negatively associated with drug spending (i.e. longer time to physician adoption leads to lower spending) and adoption extent (volume-weighted percent of physicians prescribing sitagliptin > 1 in the first 12 months) to be positively associated with anti-diabetic drug spending (i.e. a larger share of physician adoption leads to higher spending). In order to account for heterogeneous estimation using different random seed numbers in the finite mixture modeling approach, we ran the modeling 100 times utilizing randomly generated seeds, and then averaged across all the beta coefficients and standard errors to obtain the final result.

Statistical analyses were conducted using SAS software version 9.3 (SAS Institute, Cary, NC) and R software version 3.2.

## Results

### Medicare and Medicaid study sample characteristics

Table 1 shows the characteristics of the 125,264 PA Medicare, and 54,098 PA Medicaid enrollees with type II diabetes. Average age in the Medicare sample was 72, while, as expected, the Medicaid sample was relatively younger, with an average age of 50. Both samples had very similar gender breakdowns, with close to 60% being female. While the Medicare sample was 85% white, the Medicaid sample was more diverse, with 50% being white, 30% black, and 15% Hispanic. Additionally, regarding eligibility, 38% of the Medicare sample was dually eligible for Medicaid, while nearly three quarters (73%) of the Medicaid sample was enrolled through Supplemental Security Income eligibility. Regarding general health status, the Medicare sample had an average Elixhauser Index of 5.6, indicated high levels of co-morbidity. Similarly, the Medicaid sample had an average Elixhauser Index of 4.7, which, while nominally lower, still indicates the presence of multiple comorbidities. Lastly, the two samples had relatively distinct anti-diabetic drug use. In Medicare nearly two-thirds (64%) of the study sample was filling prescriptions for oral anti-diabetic medications only, 16% were using insulin or a non-insulin injectable drug only (e.g. exenatide and liraglutide), and 19% were filling prescriptions for both oral and injectable drugs. In Medicaid, 54% of the sample used oral anti-diabetic drugs only, 18% filled only prescriptions for insulin or an injectable drug, and 28% filled prescriptions for both oral and injectable drugs. Overall, while the two samples diverged in many of their demographic and clinical characteristics, all differences were largely expected, and were due to the distinct eligibility requirements of each program.

**Table 1:** Demographic Characteristics of Medicare and Medicaid Study Samples (2011)

Characteristic	Medicare (*N* = 125,264)	Characteristic	Medicaid (*N* = 50,836)
Age (Mean, SD)	72.1 (12.0)	Age (Mean, SD)	50.2 (10.1)
Female (N, %)	74,427 (59.4)	Female (N, %)	31,038 (61.1)
Race/Ethnicity (N, %)		Race/Ethnicity (N, %)	
White	105,987 (84.6)	White	25,498 (50.2)
Black	11,481 (9.2)	Black	15,341 (30.2)
Hispanic	4622 (3.7)	Hispanic	7476 (14.7)
Other race	3174 (2.5)	Other race	2521 (5.0)
Eligibility Type (N,%)		Eligibility Type (N, %)	
Dual Eligible	47,607 (38.1)	General Assistance	6655 (13.1)
Low Income Subsidy	56,358 (44.9)	Supplemental Security Income	38,076 (74.9)
Disabled	24,910 (19.9)	TANF^a^	5720 (11.3)
Type of Drug use (N,%)		Type of Drug use (N, %)	
Oral drug only	80,652 (64.4)	Oral drug only	27,436 (54.0)
Injectable drug only	20,336 (16.2)	Injectable drug only	9021 (17.8)
Combination Treatment	24,276 (19.4)	Combination Treatment	14,379 (28.3)
Elixhauser (Mean, SD)	5.6 (2.9)	Elixhauser (Mean, SD)	4.7 (2.7)
Drug Spending (Mean, SD)	$1340 ($1764)	Drug Spending (Mean, SD)	$1291 ($1881)

^a^*TANF* Temporary Assistance for Needy Families

Data sources: Medicare data from CMS, Medicaid data from PADHS

### Anti-diabetic drug spending

Unadjusted average per capita spending on anti-diabetic drugs was $1340 (SD $1764) in Medicare and $1242 (SD $1844) in Medicaid (Table 1). Figure 1 shows the non-transformed and log-transformed distributions and density plots for anti-diabetic drug spending for both the Medicare and Medicaid study samples.

### Physician adoption of Sitagliptin

A total of 7614 PA physicians prescribed anti-diabetic drugs in our study sample. The number of physicians who prescribed anti-diabetic drugs in each county varied from seven to 1136. (Additional file 1: Appendix F).

**Fig. 2:**
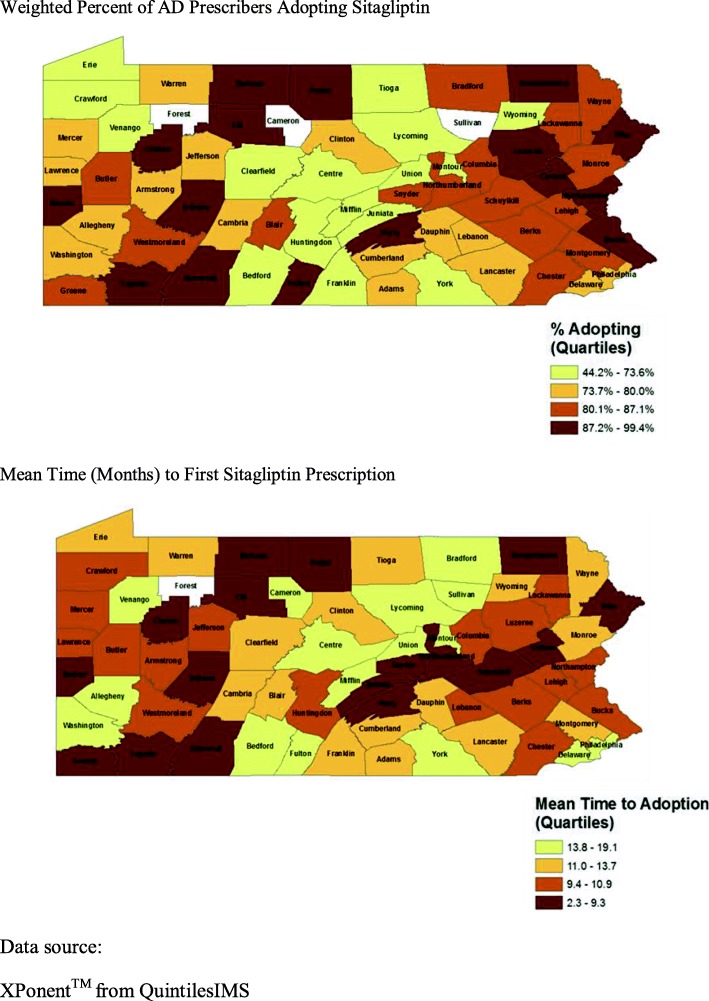
Measures of Sitagliptin Adoption by Pennsylvania County

Both the adoption time (mean time to first sitagliptin prescription), and adoption extent (percent of physicians prescribing sitagliptin at least once in its first 12 months) measures showed high variability across the counties (Fig. 2). Overall, average time to first prescription of sitagliptin was slightly less than a year (11.2 ± 3.5 months), though the time did vary markedly by county from 2.3 months *(Potter County)* to 19.1 months *(Mifflin County)*. Average weighted fraction of physicians in each county prescribing sitagliptin at least once in the first 12 months of market availability was 78% ± 12%. Again, there was substantial variation between the counties from 44% of physicians adopting *(Venango County)* to 99% of physicians adopting *(Elk County)*.

### Characteristics of subgroups

In Medicare, 55% of beneficiaries were categorized into the component with lower mean anti-diabetic drug spending, and 45% was categorized into the component with higher mean anti-diabetic drug spending (Additional file 1: See Appendix E). The largest difference in observable characteristics between the two spending components was in the type of anti-diabetic drugs used, with 85% of beneficiaries in the lower spending component utilizing oral drugs only vs. 38% of beneficiaries in the higher spending component.

Similarly, 57% of Medicaid beneficiaries were categorized in the component with lower mean anti-diabetic drug spending, while 43% were in the component with higher mean anti-diabetic drug spending (Additional file 1: See Appendix E). Again, the largest difference in observable characteristics between the two spending components was in the type of anti-diabetic drugs used, with 66% of enrollees in the lower spending component utilizing oral drugs only, while only 38% of enrollees in the higher spending component utilizing oral drugs only.

### Association of Sitagliptin Adoption with overall drug spending

For the Medicare study sample, the finite mixture model results indicated that having a higher percent of physicians within a county adopting sitagliptin was associated with higher annual anti-diabetic drug spending on average (Table 2). The magnitude, and variation, of this result differed between the two spending components. For example, a 10% increase in the number of physicians within a county adopting sitagliptin was associated with an average increase of 3.5% (95% CI: 2.0–4.9) in annual per capita anti-diabetic drug spending for the lower spending component. That same 10% increase was associated with a smaller, and non-statistically significant, average increase of 1.5% (95% CI: − 3.6 – 6.5) in anti-diabetic drug spending in the higher spending component. In comparison, mean time to first prescription of sitagliptin was found to have no statistically significant association with drug spending in either spending component.

**Table 2:** Results from the Finite Mixture Model of Anti-diabetic Drug Spending in the Medicare Study Sample (2011)

Medicare Characteristic	Spending Component					
	Low			High		
	Average Beta Coefficient	Average Standard Error	95% CI Standard Error	Average Beta Coefficient	Average Standard Error	95% CI Standard Error
Intercept	7.480	0.069	[7.345, 7.615]	7.396	0.310	[6.789, 8.004]
Time to Sitagliptin Adoption^a^	0.001	0.003	[− 0.005, 0.006]	− 0.003	0.006	[− 0.016, 0.010]
% Adopting Sitagliptin^a^	0.345	0.072	[0.203, 0.487]	0.148	0.258	[−0.359, 0.654]
Age	− 0.002	0.000	[− 0.003, − 0.001]	0.001	0.001	[− 0.001, 0.003]
Female	− 0.033	0.011	[− 0.054, − 0.012]	−0.015	0.015	[−0.044, 0.013]
Race (Ref = White)						
Black	−0.272	0.027	[−0.325, − 0.218]	−0.443	0.017	[−0.475, − 0.410]
Hispanic	−0.218	0.030	[−0.277, − 0.160]	−0.257	0.033	[−0.321, − 0.192]
Race other	−0.072	0.039	[−0.149, 0.004]	0.018	0.036	[−0.052, 0.088]
Eligibility						
Dual Eligible	−0.006	0.022	[−0.048, 0.037]	− 0.029	0.028	[− 0.084, 0.027]
Low Income Subsidy	0.202	0.021	[0.161, 0.243]	0.305	0.025	[0.255, 0.354]
Disabled	−0.146	0.018	[−0.182, − 0.110]	−0.113	0.018	[−0.148, − 0.077]
Drug Type (Ref = Combo)						
Oral only	−2.252	0.013	[−2.278, −2.226]	−2.108	0.017	[−2.142, − 2.074]
Injection only	− 0.178	0.017	[− 0.211, − 0.145]	−0.260	0.023	[−0.305, − 0.216]
Elixhauser	−0.012	0.002	[−0.016, − 0.008]	−0.007	0.003	[−0.012, − 0.002]

Data sources: Medicare data from CMS, Medicaid data from PADHS, XPonent™ from QuintilesIMS

^a^Adoption variables measured in XPonent™ from QuintilesIMS

The results for the Medicaid study sample were similar in average magnitude to the Medicare results. For example, a 10% increase in the number of physicians within a county adopting sitagliptin was associated with a smaller, and non-statistically significant average increase of 2.9% (95% CI: − 0.4 – 6.3) in annual per capita anti-diabetic drug spending for the lower spending component. That same 10% increase was associated with a significant average increase of 5.3% (95% CI: 0.3–10.3) in anti-diabetic drug spending for the higher spending component. Again, and similarly to Medicare, mean time to first prescription of sitagliptin was not statistically significantly associated with drug spending in either component (Table 3).

**Table 3:** Results from the Finite Mixture Model for Medicaid Study Sample (2011)

Medicaid Characteristic	Spending Component					
	Low			High		
	Average Beta Coefficient	Average Standard Error	95% CI Standard Error	Average Beta Coefficient	Average Standard Error	95% CI Standard Error
Intercept	6.355	0.195	[5.973, 6.736]	6.283	0.265	[5.765, 6.802]
Time to Sitagliptin Adoption^b^	0.294	0.170	[−0.039, 0.627]	0.529	0.255	[0.029, 1.028]
% Adopting Sitagliptin^b^	0.005	0.006	[−0.006, 0.017]	−0.004	0.006	[−0.016, 0.007]
Age	0.018	0.001	[0.016, 0.020]	0.019	0.001	[0.017, 0.022]
Female	−0.004	0.020	[−0.044, 0.036]	0.019	0.022	[−0.024, 0.062]
Race (Ref = White)						
Black	−0.252	0.026	[−0.303, − 0.200]	−0.414	0.032	[−0.476, − 0.352]
Hispanic	− 0.073	0.029	[− 0.129, − 0.017]	0.224	0.039	[0.147, 0.302]
Race other	0.124	0.049	[0.027, 0.220]	−0.093	0.052	[−0.194, 0.008]
Eligibility						
General Assistance	−0.287	0.031	[−0.348, − 0.227]	−0.320	0.031	[−0.381, − 0.258]
TANF^a^	− 0.256	0.033	[− 0.320, − 0.191]	−0.273	0.038	[−0.348, − 0.197]
Waiver	− 0.662	0.100	[− 0.859, − 0.466]	−0.702	0.155	[−1.006, − 0.399]
Drug Type (Ref = Combo)						
Oral Drug Only	−3.360	0.023	[−3.405, −3.314]	− 3.113	0.025	[−3.162, − 3.063]
Injectable Drug Only	−0.089	0.030	[−0.148, − 0.031]	−0.125	0.032	[−0.188, − 0.062]
Elixhauser	− 0.019	0.004	[− 0.026, − 0.011]	−0.009	0.004	[−0.017, − 0.002]

^a^*TANF* Temporary Assistance for Needy Families

Data sources: Medicare data from CMS, Medicaid data from PADHS, XPonent™ from QuintilesIMS

^b^Adoption variables measured in XPonent™ from QuintilesIMS

## Discussion

Our study reports three key findings. First, we found substantial county-level variation in both the time to adoption and in the proportion of physicians adopting sitagliptin in PA. Second, we found that the extent of physicians adopting sitagliptin was associated with higher anti-diabetic drug spending in both Medicare and Medicaid although effect sizes were relatively small. Third, we found that the distributions of anti-diabetic drug spending in the Medicaid and Medicare populations were remarkably similar, as were the magnitudes of the associations between sitagliptin adoption and drug spending in spite of differences in population characteristics and the administration of drug benefits in the two programs.

The high variability in physician adoption rates of sitagliptin by county in both average time to first prescription (2.3–19.1 months) and share of physicians prescribing sitagliptin (44 to 99% of physician) is consistent with previous findings. Studies have shown that physicians’ take up new brand name drugs at different rates, and that the proportion of brand name versus generic drug use can vary across geographic regions [3–5, 31–34]. Physician adoption of new drugs is likely influenced by many factors including practice setting (e.g. group vs. solo practice) [19, 35, 36], specialty [17, 22, 23], exposure to pharmaceutical promotion [6, 18, 21, 35], and even physician social networks [20].

Furthermore, while prior studies have highlighted the variation in physician drug adoption, our study is one of the first to show an association between the speed and extent of physician adoption of a new drug and prescription drug spending. This finding is consistent with the literature showing the diffusion of health care technologies is one of the main drivers of health care cost growth [37]. The impact of technological advancement on increased health care spending is perhaps nowhere more evident than with prescription drugs. For example, the recent double-digit annual growth rate in prescription drug spending from 2013 to 2014 has largely been attributed to the introduction of new prescription drugs [38]. Growth in prescription drug spending has also coincided with an increasing number of new drugs gaining FDA approval annually, which reached a recent peak in 2015 with 45 new drugs entering the market [39], and underscores the on-going role that new drug adoption will likely play in health care spending. Although the magnitude of the effect of sitagliptin adoption on spending was relatively small in the anti-diabetic drug class, our findings point to a potential mechanism underlying the geographic variation in prescription drug spending, namely, differences in diffusion of new drugs at the local-level. This finding could lead to interventions by payers looking to improve the efficiency of prescription drug use, by combining information on the new drug adoption behavior of physicians with information on the clinical value of new drugs, and ultimately targeting physicians for interventions such as academic detailing [40].

A strength of our study was the ability to investigate the association between physician drug adoption on prescription drug spending in both Medicare and Medicaid. This shows a more complete picture of the role of physician drug adoption since these two payers serve distinct patient populations, and have structural differences in benefit design and formulary policy. Interestingly, even though the Medicare and Medicaid study populations differed in fundamental ways such as average age (72 vs. 50 years old), racial composition (85% vs. 50% white), and average Elixhauser comorbidity index (5.6 vs. 4.7), the overall distribution of anti-diabetic drug spending in each program was remarkably similar (Fig. 1). This similarity is surprising not only due to differences in patient populations served, but also due to differences in benefit design and cost-containment tools used in the two programs. For example, Medicare plans use tiered formularies, prior authorization, and patient cost sharing to steer patients to drugs for which Prescription Drug Plans (PDPs) have negotiated larger rebates [41]. In contrast, Medicaid programs participate in the Medicaid Drug Rebate Program, which requires broad coverage of medications, and use prior authorization tools, but impose no patient cost sharing [42]. Though we did not limit our physician study sample by type of payment received, one possibility for the similar spending patterns between the two programs is that the same physicians are serving both patient populations, and their prescribing patterns remain generally stable across payers. Interestingly, the key driver of whether enrollees were in the high or low spending component was type of anti-diabetic drug use. Subjects treated with an oral anti-diabetic drug were much more likely to be assigned to the lower spending component, while subjects treated with an injectable anti-diabetic drug such as insulin were much more likely to be assigned to the higher spending component. Additionally, it is likely that the patients in the higher spending component have more severe or uncontrolled diabetes and have already failed first line oral treatment options, thus giving their treating physician multiple options in how to escalate their care, either by adding multiple oral drugs to their treatment plan, or moving on to injectable insulin. That insulin is a key driver of anti-diabetic drug spending could partially explain the relatively small effect size of sitagliptin adoption on spending, and is consistent with a recent study that found that, in Medicaid, reimbursement prices for intermediate acting insulins have grown 284% from 2001 to 2014, and by 455% for premixed insulins in the same time period [43].

Our study has several limitations. First, this study was conducted in one state, and even though PA has been shown to track closely with national averages in measures of age, gender, educational attainment, income, and measures of health care utilization, our findings might not be nationally representative [44–46]. Second, we investigated the impact of one new drug within one chronic disease drug class; in light of the fact that multiple therapeutic options exist within the diabetes drug class, and the choice set changes over time as new drugs enter the market, our results regarding sitagliptin might not generalize to the drug class as a whole. Additionally, these results might not be generalizable to other unique disease conditions. Third, like other studies using claims data, our drug spending measures do not include rebates negotiated by Part D plans in Medicare, rebates provided under the Medicaid drug rebate program, or any differences in charges by pharmacies that might be owned by managed care companies. Thus our spending measures reflect an over-estimation of the true spending amount [47]. Lastly, since there is no gold standard on how physician adoption of new prescription drugs should be measured, we defined adoption through time to first prescription, and the proportion of physicians adopting sitagliptin weighted by prescribing volume, both of which have been utilized in past studies that have investigated physician up-take of new drugs [9, 21–23, 48, 49]. Other measures of physician adoption exist, such as measures that take the share of prescriptions written for a new drug into account [50], and could strengthen the findings.

## Conclusion

This study represents the first analysis that aims to better understand regional variation in physician adoption of a newer anti-diabetic brand name prescription drug, and to determine that higher physician drug adoption is associated with higher prescription drug spending. Future research should focus on examining this association in other drug classes, and on further elucidating the underlying mechanisms surrounding why some physicians adopt brand name drugs faster than others.

## Supplementary information

**Additional file 1.** This file includes all supplemental data/tables referenced in the manuscript.

## Data Availability

The data that support the findings of this study are available from Centers for Medicare and Medicaid Services, PADHS, and Quintile’s IMS but restrictions apply to the availability of these data, which were used under license for the current study, and so are not publicly available. Data are however available from Centers for Medicare and Medicaid Services and Quintile’s IMS for a fee and under the data use agreement provisions. The websites and instructions on how others may access the relevant data are made available below: For Medicare and Medicaid enrollment and claims data: *ResDAC* (*Research Data Assistance Center*), https://www.resdac.org/ *Centers for Medicare and Medicaid Services*, https://www.cms.gov/Research-Statistics-Data-and-Systems/Research-Statistics-Data-and-Systems.html For Xponent, HCOS, and AMA masterfile data: *Quintile’s IMS* (*now IQVIA*), https://www.iqvia.com/locations/united-states

## Abbreviations

AD: Anti-diabetic
BIC: Bayesian information criterion
CCW: Chronic Condition Data Warehouse
CMS: Centers for Medicare & Medicaid Services
LIS: Low-income subsidy
NDC: National Drug Code
PA: Pennsylvania
PADHS: Pennsylvania Department of Human Services
PDE: Part D Event
TANF: Temporary Assistance for Needy Families enrollment
