# Supplementary material for: Association between physician adoption of a new oral anti-diabetic medication and Medicare and Medicaid drug spending
Source: BMC Health Serv Res. 2019 Oct 16;19:703. doi: 10.1186/s12913-019-4520-4 (PMC6794771; doi:10.1186/s12913-019-4520-4)
Supplement: Supplementary file 1 — Additional file 1. This file includes all supplemental data/tables referenced in the manuscript. [file 12913_2019_4520_MOESM1_ESM.docx]

| Additional file 1: Appendix A. List of all Anti-Diabetic Drugs included in Study | |  |
| --- | --- | --- |
| **Drug Name** | **Active Ingredient** | **Drug Sub-Class** |
| Acarbose | Acarbose | Alpha-glucosidase inhibitors |
| Precose | Acarbose | Alpha-glucosidase inhibitors |
| Cycloset | Bromocriptine Mesylate | Dopamine-2 Agonists |
| Chlorpropamide | Chlorpropamide | Sulfonylureas |
| Byetta | Exenatide | Incretin mimetics |
| Amaryl | Glimepiride | Sulfonylureas |
| Glimepiride | Glimepiride | Sulfonylureas |
| Glipizide | Glipizide | Sulfonylureas |
| Glipizide Er | Glipizide | Sulfonylureas |
| Glipizide Xl | Glipizide | Sulfonylureas |
| Glucotrol | Glipizide | Sulfonylureas |
| Glucotrol Xl | Glipizide | Sulfonylureas |
| Glipizide-Metformin | Glipizide/Metformin Hcl | Oral combination therapy |
| Diabeta | Glyburide | Sulfonylureas |
| Glyburide | Glyburide | Sulfonylureas |
| Glyburide Micronized | Glyburide,Micronized | Sulfonylureas |
| Glynase | Glyburide,Micronized | Sulfonylureas |
| Glucovance | Glyburide/Metformin Hcl | Oral combination therapy |
| Glyburide-Metformin Hcl | Glyburide/Metformin Hcl | Oral combination therapy |
| Humulin 70-30 | Hum Insulin Nph/Reg Insulin Hm | Insulin |
| Novolin 70-30 | Hum Insulin Nph/Reg Insulin Hm | Insulin |
| Novolin 70-30 Innolet | Hum Insulin Nph/Reg Insulin Hm | Insulin |
| Novolog | Insulin Aspart | Insulin |
| Levemir | Insulin Detemir | Insulin |
| Lantus | Insulin Glargine,Hum.Rec.Anlog | Insulin |
| Lantus Solostar | Insulin Glargine,Hum.Rec.Anlog | Insulin |
| Apidra | Insulin Glulisine | Insulin |
| Apidra Solostar | Insulin Glulisine | Insulin |
| Humalog | Insulin Lispro | Insulin |
| Humalog Mix 50-50 | Insulin Npl/Insulin Lispro | Insulin |
| Humalog Mix 75-25 | Insulin Npl/Insulin Lispro | Insulin |
| Humulin R | Insulin Regular, Human | Insulin |
| Novolin R | Insulin Regular, Human | Insulin |
| Novolog Mix 70-30 | Insuln Asp Prt/Insulin Aspart | Insulin |
| Tradjenta | Linagliptin | Dipeptidyl peptidase 4 (DPP-4) inhibitor |
| Victoza 2-Pak | Liraglutide | Incretin mimetics |
| Victoza 3-Pak | Liraglutide | Incretin mimetics |
| Fortamet | Metformin Hcl | Biguanides |
| Glucophage | Metformin Hcl | Biguanides |
| Glucophage Xr | Metformin Hcl | Biguanides |
| Glumetza | Metformin Hcl | Biguanides |
| Metformin Hcl | Metformin Hcl | Biguanides |
| Metformin Hcl Er | Metformin Hcl | Biguanides |
| Riomet | Metformin Hcl | Biguanides |
| Glyset | Miglitol | Alpha-glucosidase inhibitors |
| Nateglinide | Nateglinide | Meglitinides |
| Starlix | Nateglinide | Meglitinides |
| Humulin N | Nph, Human Insulin Isophane | Insulin |
| Novolin N | Nph, Human Insulin Isophane | Insulin |
| Novolin N Innolet | Nph, Human Insulin Isophane | Insulin |
| Actos | Pioglitazone Hcl | Thiazolidinediones (TZD) |
| Actoplus Met | Pioglitazone Hcl/Metformin Hcl | Oral combination therapy |
| Actoplus Met Xr | Pioglitazone Hcl/Metformin Hcl | Oral combination therapy |
| Duetact | Pioglitazone/Glimepiride | Oral combination therapy |
| Symlin | Pramlintide Acetate | Amylin Analogue |
| Symlinpen 120 | Pramlintide Acetate | Amylin Analogue |
| Symlinpen 60 | Pramlintide Acetate | Amylin Analogue |
| Prandin | Repaglinide | Meglitinides |
| Prandimet | Repaglinide/Metformin Hcl | Oral combination therapy |
| Avandia | Rosiglitazone Maleate | Thiazolidinediones (TZD) |
| Avandaryl | Rosiglitazone/Glimepiride | Oral combination therapy |
| Avandamet | Rosiglitazone/Metformin Hcl | Oral combination therapy |
| Onglyza | Saxagliptin Hcl | Dipeptidyl peptidase 4 (DPP-4) inhibitor |
| Kombiglyze Xr | Saxagliptin Hcl/Metformin Hcl | Oral combination therapy |
| Janumet | Sitagliptin Phos/Metformin Hcl | Oral combination therapy |
| Januvia | Sitagliptin Phosphate | Dipeptidyl peptidase 4 (DPP-4) inhibitor |
| Tolazamide | Tolazamide | Sulfonylureas |
| Tolbutamide | Tolbutamide | Sulfonylureas |

Appendix B. Study Flow Chart of Anti-Diabetic Prescribers in Pennsylvania

Physicians prescribing AD medications during 2007-2011.  **(N=31,823)**

Exclude those who (1) are out of state and then (2) do not have age, gender information during 2007-2011. **(N=9,969)**

AD prescribers who were in PA and have age, gender information during 2007-2011. **(N=21,854)**

Exclude those who do not have any affiliation(s) during 2007-2011. **(N=1,637)**

AD prescribers who have any affiliations during 2007-2011. **(N=20,217)**

Exclude those who did not have some AD prescription in each quarter of 1 year proceeding sitagliptin introduction and at least 1 AC/AH/ST prescription in each year of 2008-2011. **(N=12,603)**

AD prescribers have some AD prescription in each quarter of 1 year proceeding sitagliptin’s introduction and at least 1 AC/AH/ST prescription in each year of 2008-2011 **(N=7,614)**

*Note: AD = Anti-diabetic

AH = Anti-hypertensive

AC = Anti-coagulant

ST = Statin

Data source:

XPonent^TM^ from QuintilesIMS, HCOS

Appendix C1. Study Flow Chart of 2011 Medicare Study Sample

PA beneficiaries with ≥1 AD prescription fill in 2011 **(N=174,183)**

Exclude those who do not have at least 1 AD prescription fill in 2011 **(N=681,133)**

PA beneficiaries with full 12 month enrollment in Parts A, B, D in 2011 **(N=132,071)**

Exclude those who do not meet criteria for a type II diabetes diagnosis **(N=6,807)**

Exclude those who do not have full 12 month enrollment in Parts A, B, D in 2011. **(N=42,112)**

Total number of beneficiaries in 2011 PA Medicare ≥18 years old  **(N=855,316)**

PA beneficiaries meeting criteria for type II diabetes **(N=125,264)**

Appendix C2. Study Flow Chart of 2011 Medicaid Study Sample

PA enrollees with ≥1 AD prescription fill in 2011 **(N=70,435)**

Exclude those who do not have at least 1 AD prescription fill in 2011 **(N=1,056,688)**

PA enrollees with full 12 month enrollment in 2011 **(N=65,133)**

Exclude those who do not meet criteria for a type II diabetes diagnosis **(N=14,297)**

Exclude those who do not have full 12 month enrollment in 2011 **(N=5,302)**

Total number of enrollees in 2011 PA Medicaid: ≥18 years old, not dual eligible **(N=1,127,123)**

PA enrollees meeting criteria for type II diabetes **(N=50,836)**

| Appendix D1. Comparison of Model Fit Statistic (BIC) of Finite Mixture Models for Medicare Study Sample | | | |
| --- | --- | --- | --- |
| **Number of Components** | **Distributions** | | **BIC** |
| 1 | Normal | | 495,119 |
| 1 | Gamma | | 507,373 |
|  |  | |  |
| 2 | 2 Normal | | 457,754 |
| 2 | 2 Gamma | | 460,713 |
| 2 | 1 Normal, 1 Gamma | | 457,823 |
|  |  | |  |
|  |  | |  |
| Appendix D2. Comparison of Model Fit Statistic (BIC) of Finite Mixture Models for Medicaid Study Sample | | | |
| **Number of Components** | **Distributions** | **BIC** | |
| 1 | Normal | 227,798 | |
| 1 | Gamma | 234,073 | |
|  |  |  | |
| 2 | 2 Normal | 209,052 | |
| 2 | 2 Gamma | 208,988 | |
| 2 | 1 Normal, 1 Gamma | 209,674 | |

| Appendix E. Comparison of demographic, eligibility, and clinical characteristics between the model derived components for both the Medicare and Medicaid Study Samples | | | | | |
| --- | --- | --- | --- | --- | --- |
| **Variable** | **Medicare** | | **Variable** | **Medicaid** | |
|  | **Lower Component** | **Higher Component** |  | **Lower Component** | **Higher Component** |
| Age (Mean, SD) | 72.4 (12.0) | 72.1 (12.0) | Age (Mean, SD) | 50.1 (9.9) | 50.3 (10.3) |
| Female (%) | 59.1 | 59.8 | Female (%) | 63.3 | 58.3 |
| Race/Ethnicity (%) |  |  | Race/Ethnicity (%) |  |  |
| White | 84.4 | 84.7 | White | 50.7 | 49.4 |
| Black | 9.5 | 8.9 | Black | 29.2 | 31.5 |
| Hispanic | 3.6 | 3.9 | Hispanic | 15.1 | 14.2 |
| Other race | 2.5 | 2.6 | Other race | 5..0 | 4.9 |
| Eligibility Type (%) |  |  | Eligibility Type (%) |  |  |
| Disabled | 18.8 | 21.3 | General Assistance | 13.4 | 12.7 |
| Dual Eligible | 35.1 | 41.9 | Supplemental Security Income | 74.1 | 75.9 |
| Low Income Subsidy | 42.0 | 49.2 | TANF | 11.7 | 10.6 |
| Type of Drug use (%) |  |  | Type of Drug use (%) |  |  |
| Oral drug only | 85.8 | 37.6 | Oral drug only | 66.2 | 37.6 |
| Injectable drug only | 7.4 | 27.4 | Injectable drug only | 0 | 41.5 |
| Combination | 6.8 | 35.1 | Combination | 33.8 | 20.9 |
| Elixhauser (Mean, SD) | 5.3(2.9) | 5.6(3.0) | Elixhauser (Mean, SD) | 4.5(2.5) | 4.9(2.9) |

* TANF=Temporary Assistance for Needy Families

| Appendix F. Number of Anti-diabetic (AD) Prescribers, AD Medicare Beneficiaries, and AD Medicaid Enrollees by County | | | |
| --- | --- | --- | --- |
| County Name | Number of AD Prescribers | Number of AD Medicare Beneficiaries | Number of AD Medicaid Enrollees |
|  |  |  |  |
| Adams | 35 | 1,149 | 134 |
| Allegheny | 1,001 | 4,913 | 4,134 |
| Armstrong | 25 | 428 | 327 |
| Beaver | 70 | 784 | 586 |
| Bedford | 14 | 624 | 246 |
| Berks | 210 | 4,599 | 1,833 |
| Blair | 83 | 1,558 | 635 |
| Bradford | 42 | 1,039 | 253 |
| Bucks | 373 | 4,570 | 843 |
| Butler | 74 | 855 | 405 |
| Cambria | 87 | 1,227 | 680 |
| Cameron | 2 | 122 | 29 |
| Carbon | 25 | 1,392 | 175 |
| Centre | 68 | 1,098 | 278 |
| Chester | 236 | 3,908 | 494 |
| Clarion | 17 | 703 | 196 |
| Clearfield | 36 | 1,376 | 458 |
| Clinton | 18 | 448 | 185 |
| Columbia | 34 | 893 | 247 |
| Crawford | 41 | 1,521 | 423 |
| Cumberland | 161 | 2,231 | 358 |
| Dauphin | 174 | 1,992 | 986 |
| Delaware | 369 | 5,134 | 1,638 |
| Elk | 13 | 805 | 150 |
| Erie | 190 | 2,936 | 1,316 |
| Fayette | 59 | 1,131 | 1,202 |
| Forest | 0 | 122 | 24 |
| Franklin | 76 | 2,013 | 299 |
| Fulton | 7 | 321 | 52 |
| Greene | 16 | 401 | 314 |
| Huntingdon | 16 | 853 | 225 |
| Indiana | 41 | 579 | 349 |
| Jefferson | 18 | 875 | 281 |
| Juniata | 4 | 361 | 70 |
| Lackawanna | 142 | 3,368 | 724 |
| Lancaster | 309 | 5,618 | 1,476 |
| Lawrence | 39 | 765 | 443 |
| Lebanon | 62 | 1,471 | 384 |
| Lehigh | 253 | 4,366 | 1,266 |
| Luzerne | 168 | 5,884 | 1,122 |
| Lycoming | 55 | 1,680 | 461 |
| McKean | 14 | 879 | 194 |
| Mercer | 65 | 1,488 | 576 |
| Mifflin | 19 | 840 | 225 |
| Monroe | 65 | 2,054 | 541 |
| Montgomery | 608 | 6,547 | 1,097 |
| Montour | 29 | 193 | 55 |
| Northampton | 193 | 4,736 | 891 |
| Northumberland | 31 | 1,878 | 377 |
| Perry | 18 | 472 | 105 |
| Philadelphia | 1,136 | 12,688 | 15,972 |
| Pike | 13 | 845 | 116 |
| Potter | 4 | 284 | 72 |
| Schuylkill | 70 | 2,754 | 559 |
| Snyder | 13 | 471 | 91 |
| Somerset | 33 | 719 | 304 |
| Sullivan | 1 | 139 | 28 |
| Susquehanna | 14 | 692 | 121 |
| Tioga | 18 | 681 | 147 |
| Union | 23 | 493 | 86 |
| Venango | 21 | 878 | 251 |
| Warren | 13 | 816 | 128 |
| Washington | 114 | 1,181 | 701 |
| Wayne | 17 | 958 | 143 |
| Westmoreland | 186 | 1,708 | 1,253 |
| Wyoming | 16 | 393 | 97 |
| York | 217 | 4,364 | 1,005 |
| Total | 7,614 | 125,264 | 50,836 |
